# The antitumor effect of oncolytic respiratory syncytial virus via the tumor necrosis factor-alpha induction and ROS-bax-mediated mechanisms

**DOI:** 10.1186/s12885-023-11326-y

**Published:** 2023-08-28

**Authors:** Mehdi Samadi, Talat Mokhtari-Azad, Ahmad Nejati, Zahra Norooz-Babaei, Abbas Rahimi Foroushani, Mohammad Reza Haghshenas, Fatemeh Adjaminejad, Hedieh Zargaran, Vahid Salimi, Amir Ghaemi

**Affiliations:** 1 Virology Department, School of Public Health, Tehran University of Medical Sciences, Tehran, Iran; 2 Department of Statistics and Epidemiology, School of Public Health, Tehran University of medical sciences, Tehran, Iran; 3 Department of Influenza and Other Respiratory Viruses, Pasteur Institute of Iran, Tehran, Iran; 4 Department of Microbiology, Molecular, and Cell-Biology Research Center, Faculty of Medicine, Mazandaran University of Medical Sciences, Sari, Iran

**Keywords:** Apoptosis, Autophagy, Human respiratory syncytial virus, Human papillomavirus, Oncolytic viruses, Reactive oxygen species

## Abstract

**Background:**

Cervical cancer represents one of the most prevalent cancers among women worldwide, particularly in low- and middle-income nations. Oncolytic viruses (OVs) can infect cancer cells selectively and lethally without harming normal cells. Respiratory syncytial virus (RSV) is an oncolytic virus for anticancer therapy because of its propensity to multiply within tumor cells. This research aimed to assess the in vitro antitumor activities and molecular basis processes of the oncolytic RSV-A2 on the TC-1 cancer cells as a model for HPV‑related cervical cancers.

**Methods:**

Cellular proliferation (MTT) and lactate dehydrogenase (LDH) release assays were used to investigate the catalytic impacts of RSV-A2 by the ELISA method. Real-time PCR and flow cytometry assays were utilized to assess apoptosis, autophagy, intracellular concentrations of reactive oxygen species (ROS), and cell cycle inhibition.

**Results:**

Our MTT and LDH results demonstrated that TC-1 cell viability after oncolytic RSV-A2 treatment was MOI-dependently and altered significantly with increasing RSV-A2 virus multiplicity of infection (MOI). Other findings showed that the RSV-A2 potentially resulted in apoptosis and autophagy induction, caspase-3 activation, ROS generation, and cell cycle inhibition in the TC-1 cell line. Real-time PCR assay revealed that RSV-A2 infection significantly elevated the Bax and decreased the Bcl2 expression.

**Conclusions:**

The results indicated that oncolytic RSV-A2 has cytotoxic and inhibiting effects on HPV-associated cervical cancer cells. Our findings revealed that RSV-A2 is a promising treatment candidate for cervical cancer.

## Background

Cancer of the cervix is the 4th most common malignancy among women globally, with an anticipated 604,000 newly diagnosed cases and 342,000 fatalities in 2020 based on the most recent study on worldwide cancer statistics of 2022. Almost 90% of new deaths and cases occur in low- and middle-income nations [1, 2]. HPV (human papillomavirus) causes 95% of cervix cancer cases. HPV 16 and 18 (HPV types 16 and 18) are linked to at least 70% of cervix cancers [1, 3]. Surgery, chemotherapy, radiotherapy, or a combination may be used to treat cervical cancer [4, 5]. Nevertheless, these methods may be ineffective if cancer has already progressed. In addition, chemotherapy resistance and side effects have been significant obstacles in cervical cancer therapy [6]. Therefore, it is vital to identify novel treatments for cervical cancer. Oncolytic viruses (OVs) are proposed as a novel, effective, and safe therapeutic option among the alternative treatments.

Due to cancer cells’ failure to generate Interferon type 1 (IFN-1) signaling, OVs can replicate freely inside tumor cells, causing oncolytic and releasing viral progeny to continue the infection cycle [7, 8]. Oncolytic viruses are a family of cancer treatments that exhibit their anticancer action by destroying cancerous cells selectively and inducing antitumor immunity in the patient. They are naturally replicating or genetically engineered viruses designed to proliferate selectively in cancer cells, causing cell lysis while sparing healthy cells [9]. To date, three oncolytic viruses (OVs), including Encoring (H101) (adenovirus), River (Riga virus) (echovirus), and T-VEC (Illogic™) (herpes simplex-1 virus), have received regulatory approval for the treatment of head and neck, melanoma, and advanced melanoma cancers, respectively. Many more recombinant viruses from different families are undergoing clinical trials in patients with cancer. Oncolytic properties have been confirmed for the following viruses: Newcastle disease virus, reovirus, adenovirus, herpes simplex virus type 1, vaccinia virus, vesicular stomatitis virus, influenza virus, coxsackievirus, measles virus, and respiratory syncytial virus [10–14].

RSV (Human respiratory syncytial virus) is an enveloped, single-stranded, non-segmented, negatively charged RNA virus that belongs to the *Pneumoviridae* family and genus *Orthopneumovirus* [15–17]. RSV is a respiratory pathogen that causes various respiratory problems, including mild colds, bronchitis, and pneumonia [18, 19]. In the past, evidence of the oncolytic function of the RSV A2 strain (RSV-A2) was investigated in different tumor tissues such as the skin (A431) [20], breast [21], hepatocellular carcinoma cell (HCC) [22], and prostate cancer cells comprising prostate cancer cell line 3 (PC-3) and lymph node carcinoma of the prostate (LNCaP) [23, 24]. Previous experiments have investigated that RSV-A2 oncolytically destroys cancer cells via triggering apoptosis mechanism [20–24]. A study by Echchgadda et al. (2009) revealed that RSV-A2 can induce apoptosis via intrinsic and extrinsic pathways in PC-3 cancer cells [23]. However, there is a paucity of information regarding the oncolytic mechanism of this virus in HPV‑related cervical cancers. The eradication of tumor cells selectively is frequently dependent on the virus strain, cancer type, tumor microenvironment (TME), and the host’s immune system [7]. The current study aimed to investigate the oncolytic potential and mechanism of the RSV-A2 strain virus in the C57 mouse TC-1 cancer cell (HPV16-related cancer generating human E6/E7 oncoproteins (major transforming proteins of many types of papillomaviruses)) as HPV‑related cervical cancer model for the first time [5, 25]. In more detail, we tried to study the virus’s function in different cell death pathways, such as apoptosis, autophagy, and reactive oxygen species (ROS).

## Materials and methods

### Cell culture

TC-1 murine cell lines (producing HPV-16 E6/E7 oncoproteins and were obtained from the Cell Bank of the Pasteur Institute in Tehran, Iran.) grew in Dulbecco’s Modified Eagle Medium (DMEM) (Gibco, UK) with 10% FBS (fetal bovine serum), 2 mM L-glutamine, 25 mM 4-(2-hydroxyethyl)-1-piperazineethanesulfonic acid (HEPES), penicillin (100 U/ml) and streptomycin (100 g/ml) (pen/ strep) with 5% CO2 at 37 °C [8].

### Oncolytic RSV-A2 preparation, precipitation, and titration

Oncolytic RSV-A2 (A donation from Louis Bont to Wilhelmina Children’s Hospital, Utrecht University Medical Center, Netherlands) [20] was prepared and grown using Human epithelial cells 2 (HEp-2). The virus was precipitated using the Polyethylene glycol (PEG) technique, and its titer was determined by the plaque assay. In order to accomplish this, a tenfold RSV-A2 serial dilution was made in DMEM to infect Hep-2 cell lines with 80–90% confluency in a 24-well plate at 37 °C for 1.5 h. After virus absorption, RSV-A2-treated cells were covered with 0.5% methylcellulose in DMEM medium with 2% FBS and kept for six days at 37 °C. The methylcellulose layer was drained, and cells were fixed at room temperature for 30 min with 4% formaldehyde. Using an optical microscope, RSV-A2 plaques were counted after cells were dyed with 20% ethanol containing 0.2% crystal violet.

### Methyl thiazolyl tetrazolium (MTT) viability assay

By employing a cell proliferation analysis reagent (MTT) (Sigma, USA), the cytotoxic activity of oncolytic RSV-A2 was assessed. TC-1 cell line was cultured at 3 × 10^4^/100 µl density in each well of a 96-well plate and left to incubate for twenty-four hours. Following infection cell lines with RSV-A2 at multiplicity of infections (MOIs) of 1, 5, 10, and 15 for one hour, the media was removed and renewed with DMEM having 1% pen/strep and 1% FBS. After 72 h, 100 µl/well MTT solution was added and kept at 37 °C for three hours. Following incubation, media was removed, and 100 µl of DMSO (dimethyl sulfoxide) were used to dissolve the formazan crystals. Lastly, the MTT reduction was detected by determining its absorbance at 540 nm utilizing a microplate reader. (Anthos Labtec Instruments, Austria). Each exam was performed a minimum of three times.

### Lactate dehydrogenase (LDH) assay

The lysis of cells was evaluated by measuring the LDH enzyme release into the culture medium using a commercial LDH assay reagent under the manufacturer’s instructions (Takara Bio, Tokyo, Japan). In brief, TC-1 cancer cell lines were cultured at 3 × 10^4^ cells/100 µl density in each well of a 96-well plate and infected with oncolytic RSV-A2 at MOIs of 1, 5, 10, and 15 for one hour. The medium was then renewed and supplied with 1% pen/strep and 1% FBS for 72 h. The microplate was centrifuged at a 250 g/minute rate for ten minutes. Then, 100 µl of each well’s supernatant was transferred to a new 96-well plate, followed by adding of 100 µl of LDH test solution to each well and 30 min of room-temperature incubation. Lastly, absorption was determined at 490 nm wavelength, and calculations were performed using the formula included with the kit. Each experiment was repeated at least three times.

### Analysis of apoptosis via flow cytometry

Annexin V-fluorescein isothiocyanate/propidium iodide (Annexin V-FITC/PI) staining by flow cytometry illustrated the oncolytic RSV-A2-induced apoptosis of TC-1 cancer cell lines. Using a commercial Annexin V /PI Apoptosis Assay Kit (BD Biosciences, USA), the impact of RSV-A2 on apoptosis in TC-1 tumor cell lines was analyzed. Briefly, each well of a six-well plate was seeded with 5 × 10^5^ cells. Then, TC-1 cell lines were treated with RSV-A2 virus at MOIs of 1, 5, 10, and 15 for one hour, and the medium was replaced with DMEM with 1% pen/strep and 1% FBS for seventy-two hours. Non-infected cell lines were regarded as a control group. The TC-1 cancer cells were trypsinized and exposed to the DMEM to eliminate the effect of the trypsin. Next, 100ml of the binding buffer was appropriately mixed with 5ml of propidium iodide reagent and 5ml of FITC-conjugated anti-annexinV/PI labeling antibody (BD Biosciences, USA). After 15 min of incubation in the dark and at room temperature, the apoptotic percentage of the cells was determined. The average proportion of annexin V-stained cells versus the control group was compared in flow cytometry annexin V staining. Each test was conducted three times.

### ROS measurement

Using 2,7- dichlorofluorescein diacetate (DCFH-DA) (Sigma-Aldrich, USA), the amount of intracellular reactive oxygen species (ROS) was determined. In summary, TC-1 tumor cell lines were seeded at a 5 × 10^5^ density per well in a 6-well plate and then treated with MOI values of 1, 5, 10, and 15 for one hour. The media was replaced after 72 h, with DMEM having 1% FBS and 1% pen/strep. After one hour of incubation with DCFH-DA, the samples were trypsinized and washed in phosphate-buffered saline (PBS). Eventually, the severity of cell fluorescence was determined by the flow method (BD Biosciences, USA). Each test was carried out at least three times.

### Extraction of total RNA, cDNA synthesis, and quantitative real-time PCR analysis (qRT-PCR)

5 × 10^5^ TC-1 cancer cell lines/well were cultured in a six-well plate to analyze gene expression. Then, TC-1 cell lines were treated with oncolytic RSV-A2 virus at MOIs of 1, 5, 10, and 15 for one hour, and the medium was replaced with DMEM having 1% pen/strep and 1% FBS for seventy-two hours. Non-infected cell lines were considered the control group. According to the manufacturer’s instructions, total RNA was extracted using the High Pure Isolation Kit (Roche, Germany) from TC-1 cells. Utilizing a Transcriptor First Strand cDNA Synthesis Kit (Roche, Germany), cDNA (complementary DNA) was synthesized from total RNA by reverse transcription. The primers of the target (Bcl-2-associated X protein (Bax) and B-cell leukemia/lymphoma 2 protein (Bcl2)) and β-actin housekeeping (internal control) genes were designed with Primer-BLAST software (NCBI) considering the features of the SYBR-green real-time PCR (RT-PCR) approach (Table 1). To quantify the mRNA (messenger RNA) levels of Bax and Bcl2 genes, relative quantitative SYBR-green RT-PCR using RealQ Plus 2× Master Mix Green (Denmark) on the Rotor-Gene Q instrument (QIAGEN) was applied. The qPCR cycling conditions were set for an initial denaturation step for ten minutes at 94 °C followed by 40 amplification cycles, including denaturation for 15 s at 95 °C, annealing for 20 s at 57 °C, and an extension for 20 s at 72 °C. The amplification signals of various specimens were normalized to the β-actin cycle threshold (Ct). Then the 2^−∆∆CT^ approach was used to compare mRNA levels of infected vs. uninfected samples, which indicated a fold‑change in data analysis. Each experiment was carried out three times.

**Table 1:** Primers Used in RT- PCR Assay

*Objective genes*	*Primer sequence*
BAX	Forward:5ˊCAGAGGATGATTGCTGACGTGG 3ˊ
	Reverse: 5ˊTTAGTGCACAGGGCCTTGAGC 3ˊ
Bcl-2	Forward: 5ˊGACTTCTCTCGTCGCTACCGTC 3ˊ
	Reverse: 5ˊATCTCCCTGTTGACGCTCTCC 3ˊ
β-actin	Forward: 5ˊATGCTCCCCGGGCTGTAT 3ˊ
	Reverse: 5ˊCATAGGAGTCCTTCTGACCCATTC 3ˊ

### Enzyme-linked immunosorbent assay (ELISA) based assessment of TNF-α

The TNF-α (tumor necrosis factor-alpha) concentration was measured as an extrinsic apoptosis-promoting factor. The TNF-α ELISA kit (Abcam, USA) was utilized to determine the TNF-α concentration. For the test, TC-1 tumor cell lines were treated with an A2 strain of oncolytic RSV at various MOIs (1, 5, 10, and 15), and the tumor oncolysate was harvested 72 h later. The cell lysate was filtered through filters (40 μm) and centrifuged at 5000 rpm for ten minutes at 4 °C. After adjusting the protein content of viral tumor lysis specimens to 2 mg/ml with PBS, the TNF-α concentration was determined utilizing an ELISA kit (Abcam, USA) under the manufacturer’s protocol. Each test was carried out three times.

### Cleaved caspase-3 staining

With flow cytometry analysis, the apoptosis caused by oncolytic RSV-A2 at an MOI of 10 was quantified by the manufacturer’s instruction. To accomplish this, we targeted cleaved caspase-3, a valid indicator for dying or dead cells undergoing apoptosis. Briefly, in 6-well plates, TC-1 cancer cell lines were cultured (5 × 10^5^ cells/ well) and maintained for twenty-four hours. The tumor cell lines were infected with RSV-A2 at an MOI of 10 for one hour, and then the media was removed and renewed with DMEM with 1% pen/strep and 1% FBS for 72 h. After suspending and fixing RSV-A2-infected cells for 15 min in 4% formaldehyde, they were permeable for 10 min when treated at 24 °C with Triton X-100 (0. 2%). Then, TC-1 cells were labeled for 30 min with an anti-cleaved caspase-3 primary antibody (Cell Signaling, USA) diluted in PBS with 1% BSA (bovine serum albumin). After washing the cells with PBS, a secondary antibody labeled with PE (Anti-Donkey IgG, Bio Legend, USA) was added and left for thirty minutes at 24 °C. Flow cytometry was then employed to examine the labeled cells. (Becton & Dickinson Biosciences, USA). Each test was carried out three times.

### Detection of activated autophagy

A flow cytometer was used to assess the LC3B-II (microtubule-associated protein one light chain 3B-II) autophagosome marker using specific antibodies to identify autophagy induction. For this regard, TC-1 cell lines were seeded at 5 × 10^5^ cell lines/well density in a 6-well plate for Twenty-four hours. The cells were infected with oncolytic RSV-A2 at an MOI of 10 for one hour. Then the media was refreshed and supplied with DMEM having 1% pen/strep and 1% FBS for 72 h. After suspending and fixing RSV-A2-infected cells for 15 min in 4% formaldehyde, they were permeable for 10 min when treated at 24 °C with Triton X-100 (0. 2%). Then, TC-1 cells were labeled for 30 min using diluted anti-LC3B- II primary antibodies from Abcam (USA) in PBS with BSA (1%). Finally, a secondary Ab (antibody) (Anti-Donkey IgG, Bio Legend, USA) was used that was PE-conjugated.

### Analysis of the cell cycle

Cell cycle arrest was investigated by determining the DNA content of the nucleus after staining it with PI (propidium iodide). In summary, in 6-well plates, TC-1 cell lines were cultured at 5 × 10^5^ density of cells/well and incubated for 24 h. The cancer cell lines were infected with oncolytic RSV-A2 at an MOI of ten for one hour, and then the DMEM was removed and renewed with a medium having 1% FBS and pen/strep (1%) for seventy-two hours. The cell lines were trypsinized, centrifuged for fifteen minutes at 12,000 rpm (rotation per minute), washed twice with PBS, and fixed at -20 degrees Celsius using ice-cold 70% ethanol for three hours. After resuspending the cells in 50 µl RNase A, 50 µl PI, and 300 µl of PBS at 37 °C for thirty minutes in dark conditions. The Flow cytometer was used to perform a cell cycle distribution investigation. (Becton & Dickinson Biosciences, USA). Each experiment was carried out at least three times.

### Statistical evaluation

Graph Pad Prism (GraphPad Prism 8.4.3, CA, USA) was used to carry out all statistical evaluations. One-way ANOVA (analysis of variance) (followed by Tukey’s post-hoc analysis) and a student’s t-test (t- test).

were utilized to assess group means. **** P < 0.0001, *** P < 0.001, **P < 0.01, and *P < 0.05 were considered to be significant values.

## Results

### The plaque assay and titration

**Fig. 1:**
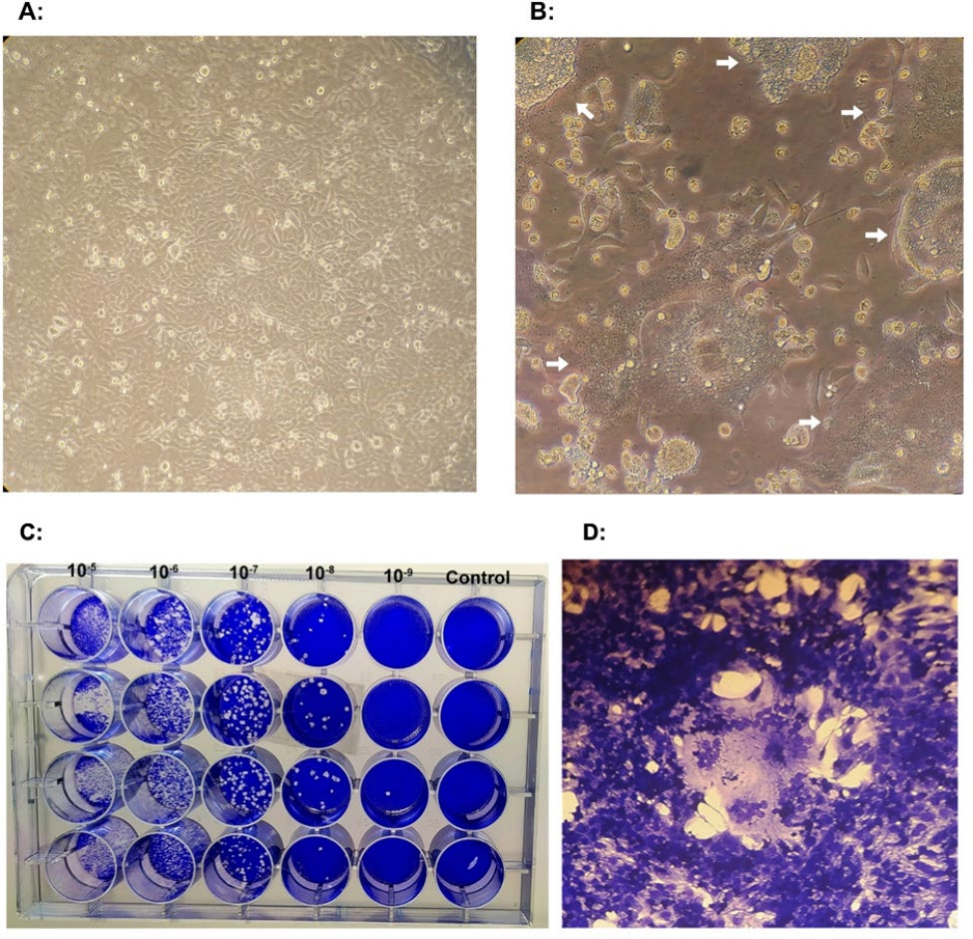
The cytopathic effect (CPE) and Plaque development caused by oncolytic RSV-A2 in HEp-2 cell culture. **A** Uninfected HEp-2 cells. **B** HEp-2 cell culture displaying RSV-A2 infection CPE (syncytia and giant multinucleated cells of various sizes, arrows) under x400 magnification and without staining **C** HEp-2 cell lines were treated with serial ten-fold dilutions of oncolytic RSV-A2 and were dyed at six days post-infection in 20% ethanol with 0.2% crystal violet. **D** Stained image of (C) at high magnification (x400) (RSV-A2 CPE).

After infecting Hep-2 cell lines with 80–90% confluency with oncolytic RSV-A2 in a 24-well plate, they were examined under a microscope on days 1, 2, 3, 4, 5, and 6 following infection. According to Fig. 1A (uninfected cells) and Fig. 1B (infected cells), RSV-A2 cytopathic effect (CPE) in the form of syncytia and giant multinucleated cells of various sizes were observed vs. the control cells. HEp-2 cells were treated with serial 10-fold dilutions of RSV-A2 for the plaque assay (Fig. 1C and D). The virus sample concentration was determined in plaque-forming units per milliliter (PFU/ml) after six days post-infection. The stock’s viral titer was 8 × 10^7^ pfu/ml.

### MTT and LDH assays for oncolytic RSV-A2 cytotoxic effect

**Fig. 2:**
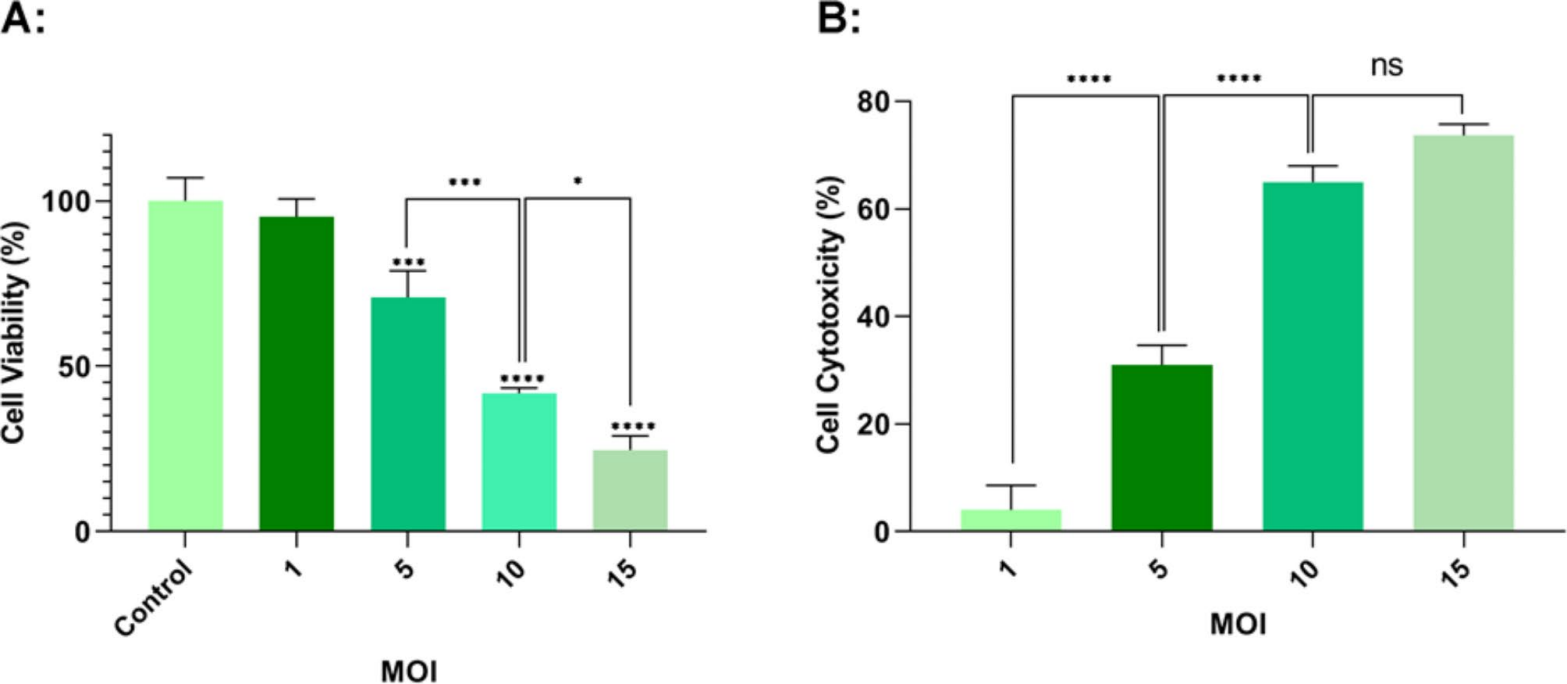
Oncolytic RSV-A2 cytotoxicity on TC-1 cell lines. **A** MTT assay to assess the cytotoxicity of oncolytic RSV-A2 on TC-1 cancer cells. TC-1 tumor cell lines were seeded and then treated with RSV-A2 at different MOIs for 72 h in 96-well plates. The results of the MTT experiment demonstrated that infection at an MOI of 15 could significantly decrease the TC-1 cell viability compared to the control. **** (P < 0.0001) and *** (P < 0.001) demonstrate a statistically significant difference among MOIs 5, 10, and 15 vs. the uninfected cells by ANOVA (one-way). * (P < 0.05) illustrates a statistically significant difference between MOI 10 in comparison to MOI 15 groups via ANOVA (one-way). **B** Oncolytic RSV-A2 Cytotoxicity on TC-1 cell lines assessed via LDH test. In 96-well plates, TC-1 tumor cells were seeded and treated with RSV-A2 at different MOIs for 72 h. The LDH data demonstrated that infections at MOIs of 5, 10, and 15 could significantly release LDH versus the control groups. However, TC-1 cancer cell lines treated with RSV-A2 at 15 MOI did not demonstrate statistically significant survivability than cultures inoculated at 10 MOI (P > 0.05). **** (P < 0.0001) represents a statistically significant difference between MOI 10 and MOI 5, as well as between the 5 and 1 groups by ANOVA(one-way).

To determine if oncolytic RSV-A2 has toxic activity on TC-1 cancer cell lines, an MTT reduction experiment was performed using RSV-A2 at different MOIs (1, 5, 10, 15). Since the MTT reduction of mitochondrial enzymes occurs only in active metabolic cells, the activity level indicates cell viability. As depicted in Fig. 2A, infection with MOIs of 10 and 15 significantly reduced the TC-1 cell viability versus the untreated group (P < 0.0001). At MOIs of 10 and 15, the TC-1 cell viability decreased to 41.80% and 24.63%, respectively. Moreover, our results showed that RSV-A2 decreased the TC-1 cancer cell viability MOI-dependently. Meanwhile, LDH (lactate dehydrogenase) is an intracellular enzyme released by necrotic tissues. Consequently, cytosolic LDH will act as an indicator for the catalytic effect of RSV-A2. The cytotoxic effect of oncolytic RSV-A2 on TC-1cells was evaluated via LDH secretion after seventy-two hours of infection at various MOIs. As shown in Fig. 2B, the highest LDH leakage, 73.67% ± 2.082, was detected seventy-two hours following treatment at an MOI of 15 in the TC-1 cancer cell lines (P < 0.0001). Though RSV-A2 treated TC-1 cell lines at 15 MOI did not demonstrate statistically significant differences in cell viability compared to those inoculated at an MOI of 10 (P > 0.05). Moreover, the findings revealed that The amount of LDH secretion elevated in TC-1 cancer cells MOI-dependently.

### Annexin V /PI analysis for determining the rate of cellular apoptosis

**Fig. 3:**
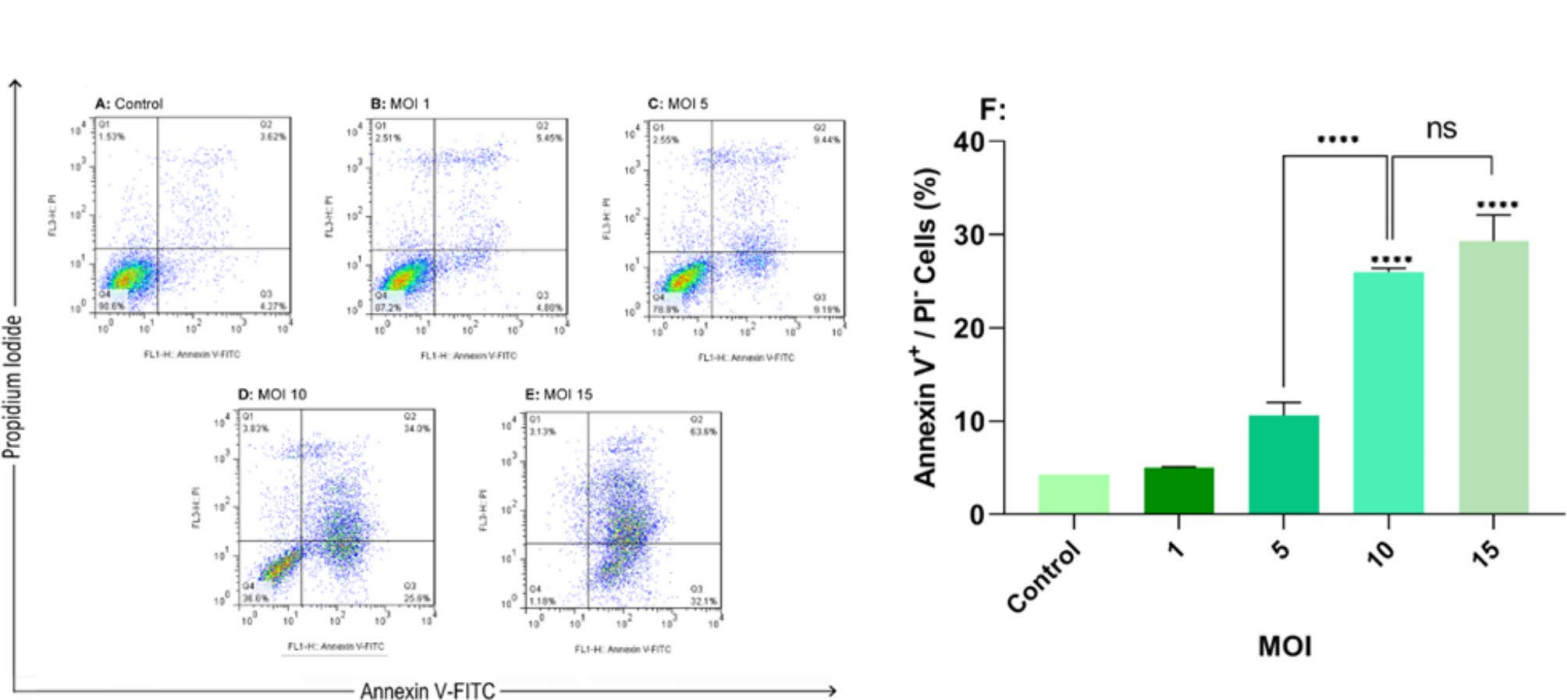
Annexin V/PI double labeling of oncolytic RSV-A2 infected TC-1 cancer cells. **A-E** TC-1 cell lines were infected with oncolytic RSV-A2 at different MOIs (1 to 15) for seventy-two hours. Then, they were labeled with annexin V/PI and evaluated with a flow cytometry method. Uninfected cells are used as a control. **F** The total percent of annexin-V-stained apoptotic cells. The findings demonstrated a statistically significant difference between infected cells (MOIs 5 to 15) and the control group. Additionally, the findings show no significant difference between MOI 10 and MOI 15 groups (p > 0.05). **** (P < 0.0001) demonstrates a statistically significant difference between infected groups (MOIs 10 and 15) in comparison to control as well as MOI 10 and 5 by ANOVA(one-way).

Flow cytometry with annexin V-FITC /PI labeling was utilized to calculate the apoptosis/ necrosis induction percentage in oncolytic RSV-A2-infected TC-1 cells. The average percentage proportion of annexin V ^+^/ PI ^−^ (early apoptosis/viable cells) in RSV-A2 treated TC-1 cancer cells at various MOIs was displayed (Fig. 3). The Highest apoptosis rate (early stage), 29.30% ± 2.800, was seen seventy-two hours post treatment at an MOI of 15 in TC-1 tumor cell lines. (P < 0.0001). While RSV-A2-treated TC-1 cells at 15 MOI did not exhibit statistical significance apoptotic activity compared to the cells infected at 10 MOI (P > 0.05). In addition, the findings demonstrated that the apoptosis induction in oncolytic RSV-A2-infected TC-1 cells increased MOI-dependently compared to the control samples.

### The effect of oncolytic RSV-A2 on intracellular ROS production

**Fig. 4:**
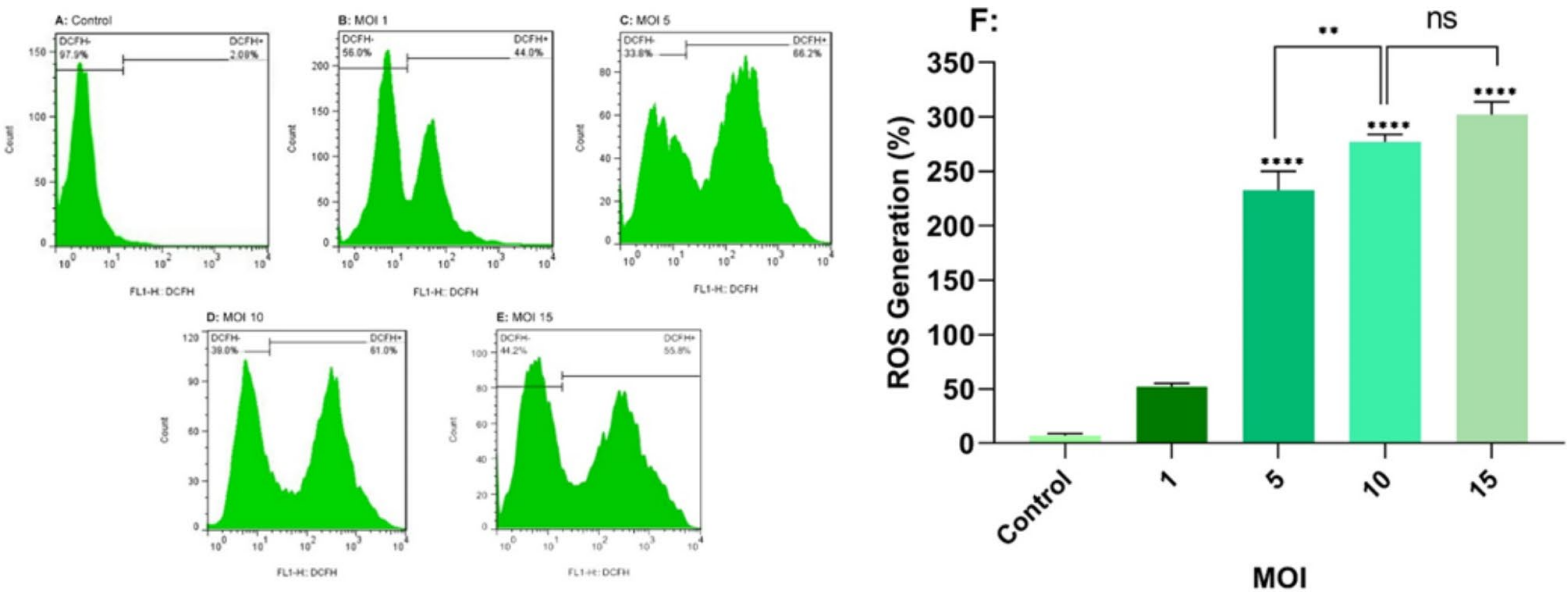
TC-1 cell lines DCFH-DA labeling after RSV-A2 treatment. **A-E** TC-1 cell lines were infected with RSV-A2 at different MOIs (1 to 15) for seventy-two hours. Untreated cells are used as a control. **F** The total percent of DCFH-DA stained apoptotic cell lines. The data demonstrated that treatment with 5, 10, and 15 MOI can significantly produce ROS compared to uninfected cells **** (P < 0.0001). While TC-1 cells inoculated with 15 MOI of RSV-A2 did not indicate statistically significant activity than cells treated with the 10 MOI (P > 0.05). ** (P < 0.01) represents a significant difference between the MOIs of the 10 and 5 groups by using ANOVA (one-way) analysis

The generation of ROS (reactive oxygen species) has been asserted to be the fundamental mechanism for several anti-tumor medicines that induce apoptotic death of cells in various cancers. We used DCFH-DA-based flow cytometry to assess intracellular ROS contents to determine whether the cell death produced by oncolytic RSV-A2 was also accompanied by elevated ROS levels in TC-1 cancer cell lines. For this, TC-1 cell lines were treated with RSV-A2 at MOIs of 1, 5, 10, and 15. As presented in Fig. 4, the highest ROS production was seen 72 h after inoculation at MOIs of 10 and 15, respectively, in TC-1 cell lines. (P < 0.0001). While TC-1 cells contaminated with 10 and 15 MOI did not exhibit statistically significant activities when compared (P > 0.05). Furthermore, RSV-A2-induced TC-1 cells produced more reactive oxygen species (ROS) versus the control cells in an MOI-dependent manner.

### Oncolytic RSV-A2 Effects on Bcl-2 and bax genes transcription

**Fig. 5:**
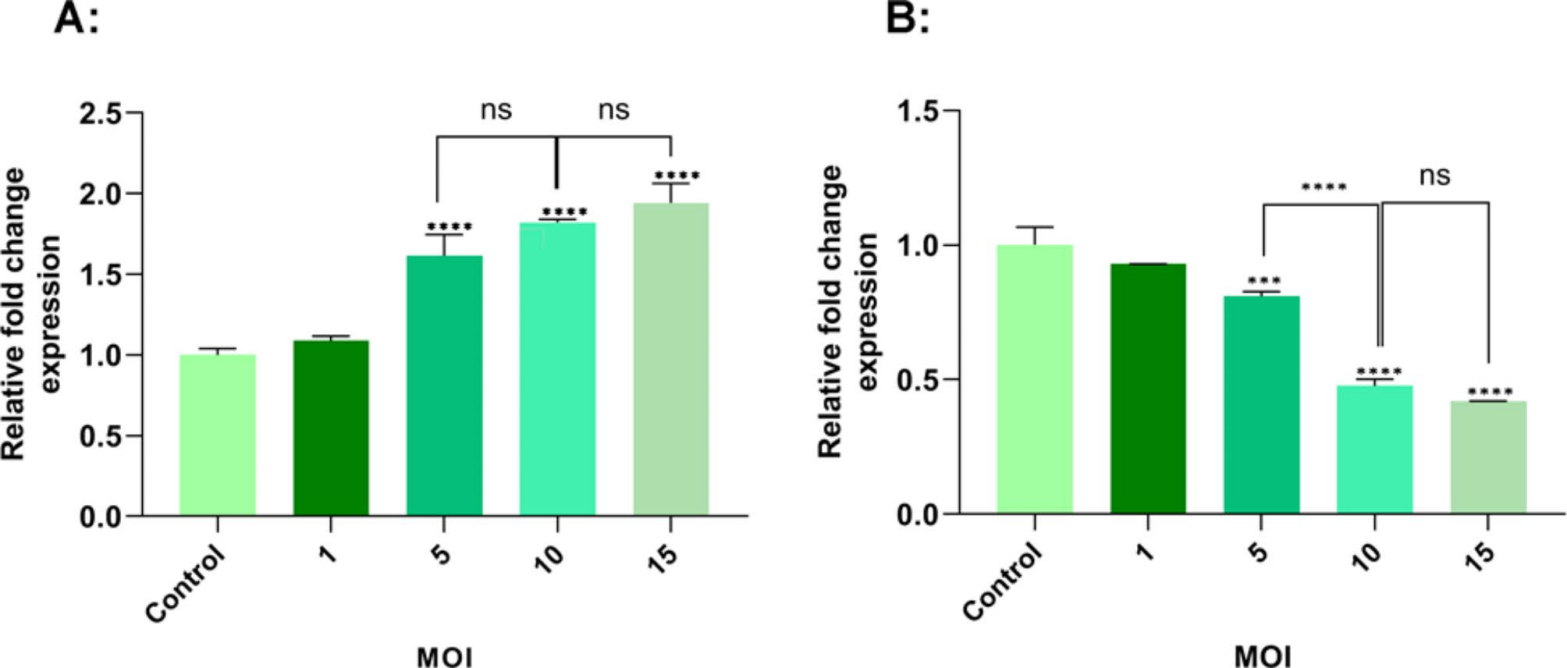
Bax and Bcl2 genes expression in RSV-A2 infected TC-1 cancer cells. TC-1 tumor cells were infected with RSV-A2 at different MOIs (1 to 15) for seventy-two hours. Then, they were analyzed for apoptotic genes (Bax & Bcl2) by Real-Time PCR. As a control, uninfected cells were utilized. **A** The results demonstrated that infection with 5,10, and 15 MOI could significantly upregulate the Bax gene expression vs. the control group (P < 0.0001). **B** The findings indicated that treatment with MOIs 10 and 15 can significantly downregulate the Bcl2 gene versus the control cells (P < 0.0001). *** (P < 0.001) shows a statistically significant Bcl2 gene expression difference between MOIs of 10 and 5 via ANOVA (one-way). ^Ns^ (P > 0.05) shows no significant difference

Following 72 h of TC-1 cancer cell lines treated with the RSV-A2 at the MOIs ranging from 1 to 15, the apoptotic gene expressions of Bax and Bcl2 were analyzed by Real-time PCR. As demonstrated in Fig. 5A, the highest Bax mRNA levels were seen at MOIs of 10 and 15, respectively, compared to the control group. (P < 0.0001). Although TC-1 cell lines infected with MOIs of 10 and 15 did not demonstrate statistically significant activity when compared (P > 0.05). In Fig. 5B, the results showed that the lowest rates of Bcl2 gene expression were seen at MOIs of 10 and 15, respectively, compared to uninfected cells. (P < 0.0001). Although infection with MOIs of 10 and 15 did not demonstrate statistically significant activity when compared (P > 0.05). Also, the level of Bcl2 gene expression in RSV-A2-infected TC-1 cells is MOI dependently vs. the uninfected cells.

### The impact of the oncolytic RSV-A2 on the protein level of TNF-α

**Fig. 6:**
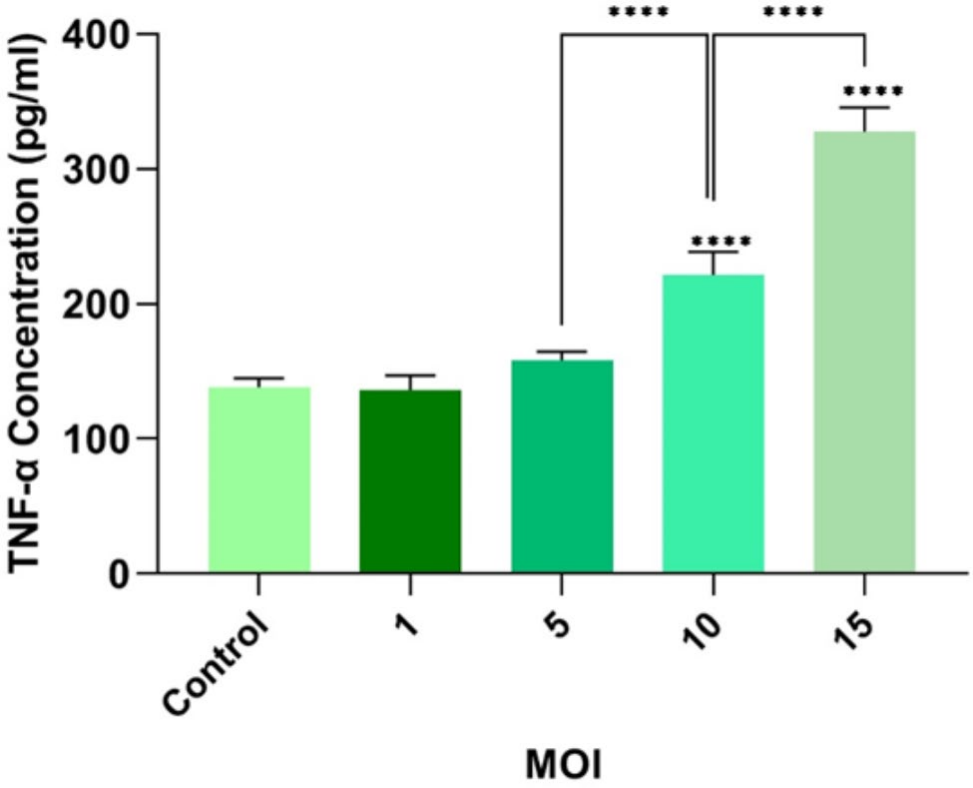
Protein level of TNF-α in the RSV-A2-infected TC-1 cells. Evaluating TNF-α in TC-1 cancer cell lines after infection with different MOIs of the RSV-A2 virus (MOI: 1, 5, 10, and 15) using the ELISA technique. Un-infected cells are considered as control groups. ****(P < 0.0001)

The protein concentration of TNF-α was assessed in the RSV-A2-infected TC-1 cells by ELISA technique. As demonstrated in Fig. 6, a significant rise in TNF-α concentration was shown in the TC-1 cell lines infected with MOIs of 10 and 15, respectively, versus the uninfected cells (P < 0.0001). These findings also indicated that the release of TNF-α as an extrinsic apoptosis biomarker during TC-1 cancer cell lysis is in an MOI-dependent way. According to the results of this study, the inhibitory concentration 50% growth (IC50) for oncolytic RSV-A2-infected TC-1 cells after 72 h post-infection was around 10 MOI. Therefore, this MOI was selected as the optimal dose (RSV-A2 IC50 MOI) for future experiments [26–28].

### Oncolytic RSV-A2 caused TC-1 cell apoptosis by caspase-3 induction

**Fig. 7:**
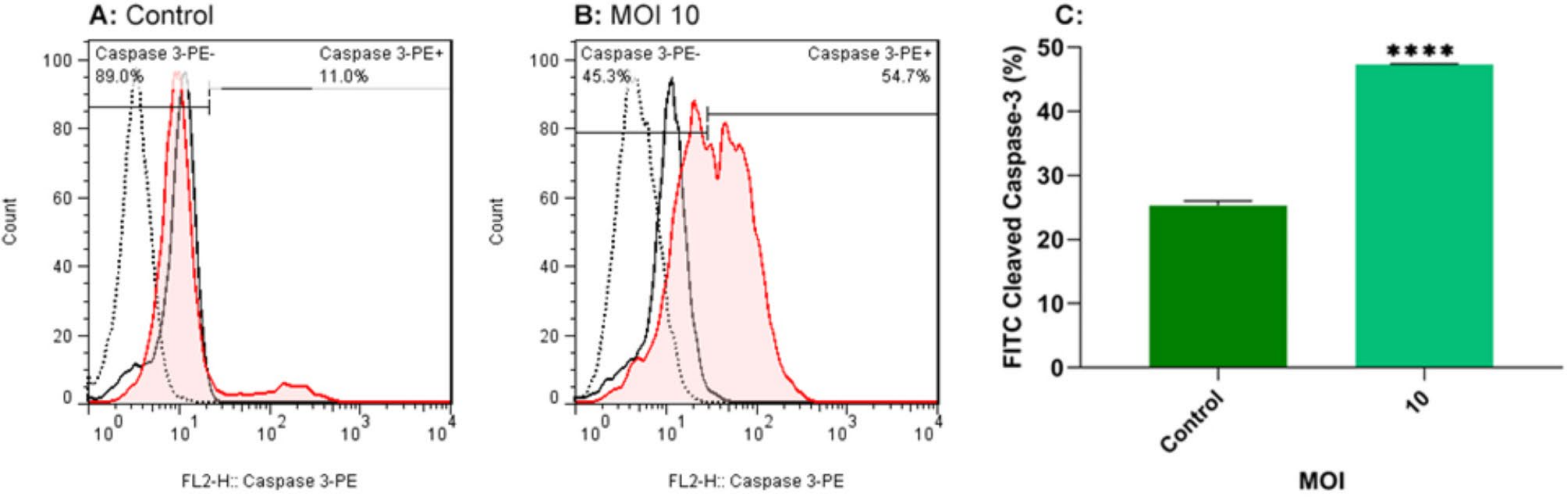
TC-1 cells stained with cleaved caspase-3 following RSV-A2 treatment. **A, B** TC-1 cell lines were treated with oncolytic RSV-A2 at 10 MOI for 72 h, stained with primary antibody (against cleaved caspase-3), stained with secondary antibodies, and subjected to flow cytometry analysis. Uninfected cell lines are utilized as a control. **C** The total percentage of cleaved caspase-3 stained apoptotic cells. Data demonstrated that inoculation at an MOI of 10 could significantly increase cleaved caspase-3 protein versus control cells. According to Student’s t-test, there is a statistically significant difference between 10 MOI and control groups (****(P < 0.0001))

Caspase-3 is a caspase effector belonging to the cysteine protease family that regulates apoptosis via intrinsic and extrinsic routes. Thus, to measure apoptosis in RSV-A2-treated cells, cleaved caspase-3 production was measured using a flow cytometry technique in the present work. RSV-A2 10 MOI was used to treat TC-1 cancer cells as the optimal oncolytic dose. As illustrated in Fig. 7, the TC-1 tumor cell lines treated with an MOI 10 significantly increased cleaved caspase-3 protein compared to control cells (P < 0.0001). These findings indicate that the virus can induce apoptosis and result in TC-1 Cell oncolytic.

### Oncolytic RSV-A2 induces autophagy in the TC-1 cancer cell lines

**Fig. 8:**
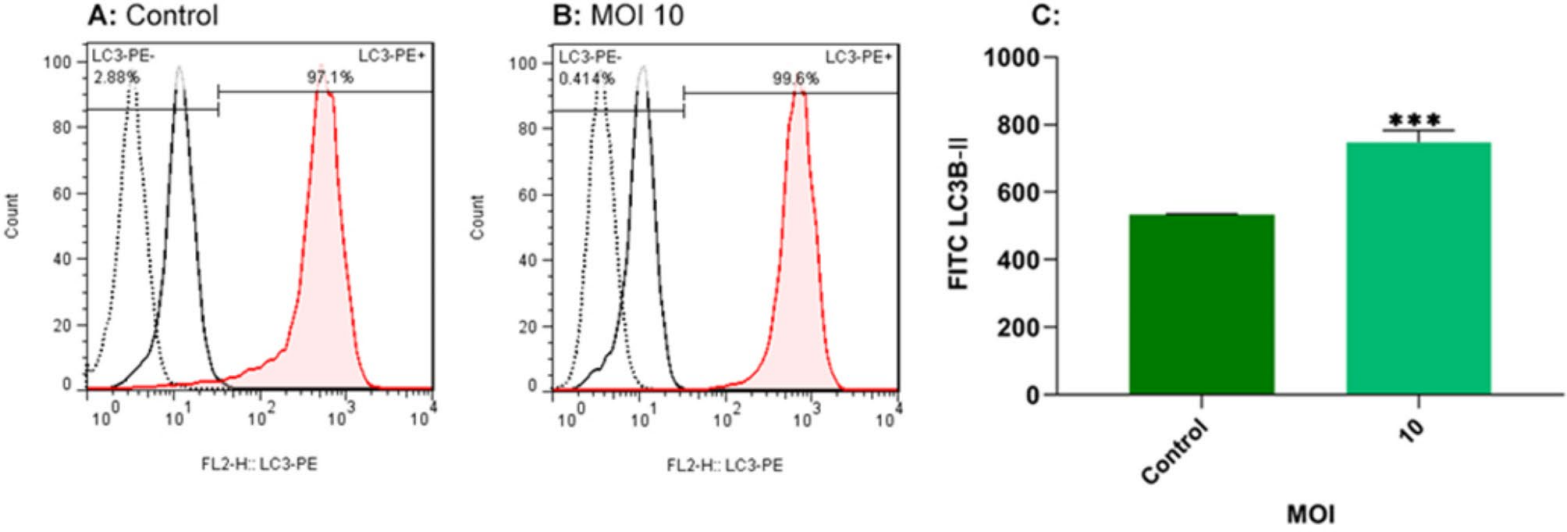
LC3B-II labeling of TC-1 cell line after RSV-A2 treatment. **A, B** TC-1 tumor cell lines were inoculated with oncolytic RSV-A2 at 10 MOI for 72 h, stained with secondary antibodies, and subjected to flow cytometric analysis. Untreated cells are used as a control. **C** The total percent of LC3B-II-stained apoptotic cells. The findings demonstrated that treatment at an MOI of 10 could significantly increase LC3-II levels vs. uninfected cells. Using Student’s t-test ***(P < 0.001) demonstrates a statistically significant difference between MOI of 10 and control cells

Autophagy is regulated by proteins like LC3B (microtubule-associated protein one light chain 3B). It is an essential protein of autophagy that undergoes a conformational change from LC3B-I to LC3B-II throughout autophagy and has distinctive functions at different stages of autophagosome construction. Seventy-two hours after infection, the LC3B-II expression was analyzed through flow cytometry in RSV-A2 infected TC-1 cells at the optimum dose of 10 MOI. As illustrated in Fig. 8, a significant rise in the protein of LC3B-II was observed in RSV-A2-induced TC-1 tumor cell lines vs. control cells (p < 0.001). These findings indicate that RSV-A2 infection causes autophagy in TC-1 cells.

### Oncolytic RSV-A2 induces sub-G1, G2, and S-phase cell cycle arrest

**Fig. 9:**
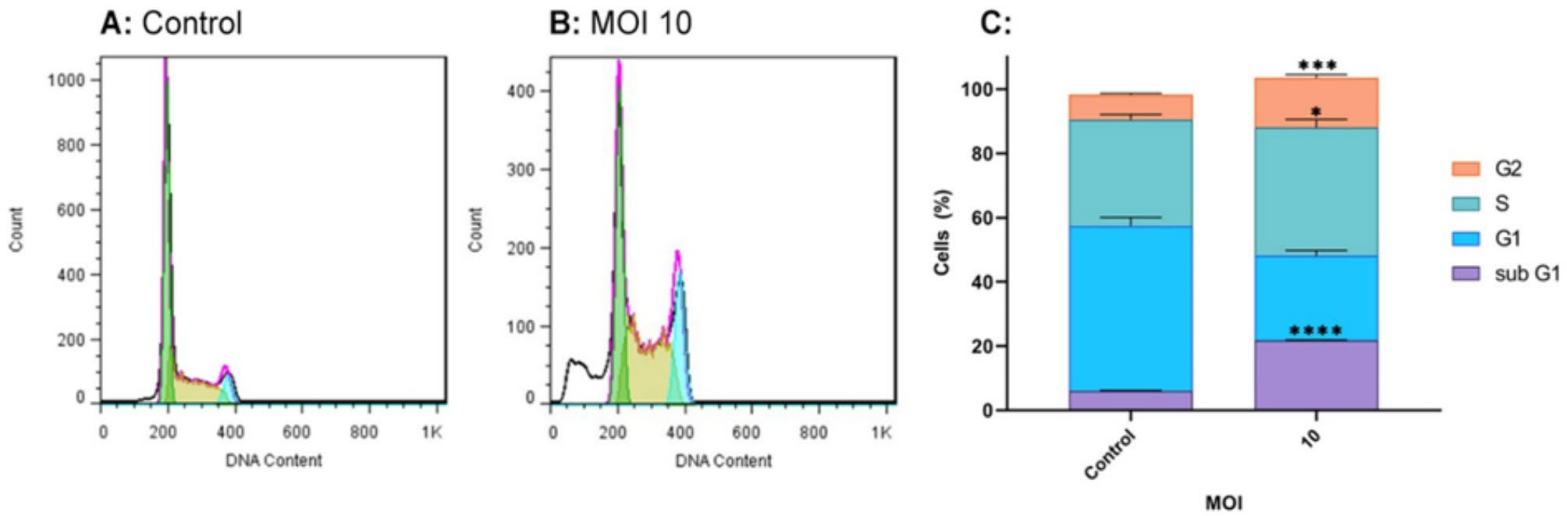
Cell cycle distribution. Differences in the Cell cycle distribution of the TC-1 tumor cell lines after adding RSV-A2 at an MOI of 10 were measured by flow cytometry for 72 hpi (hours post-infection). **A, B** PI-stained TC-1 cell lines in the sub-G1 (apoptosis), S, and G2 cell cycle phases were separated according to their DNA content. **C** The bar graph illustrates the total proportion of TC-1 cell distributions stained with propidium iodide. ****(P < 0.0001), ***(P < 0.001), and *(P < 0.05) represent statistically significant differences between MOI 10 vs. control cells using Student’s t-test

The effects of RSV-A2 on cell cycle phase distribution were investigated using flow cytometry (Becton & Dickinson Biosciences, USA) and propidium iodide as a labeling reagent. RSV-A2 IC50 MOI (10 MOI) was used to treat TC-1 cells. Figure 9 demonstrates that oncolytic RSV-A2 induces significant cell cycle arrest in oncolytic RSV-A2 infected TC-1 cells. It significantly increased the fractions of sub-G1 (sub-gap1) (apoptotic phase) (P < 0.0001), S (synthesis) (P < 0.05), and G2 (gap2) phases (P < 0.001) in RSV-A2 infected TC-1 cells vs. control cells (un-infected).

## Discussion

Oncolytic viral therapy is a promising approach to cancer treatment because it selectively attacks cancerous tumors without affecting nontransformed or normal cells [9]. There are indications that certain OVs limit the multiplication of cancerous cells and induce immunological responses, resulting in the eventual death of malignant cells [29]. Multiple studies have demonstrated that RSV-A2, an oncolytic virus, positively affects various human cancers, such as skin (A431) [20], breast [21], hepatocellular carcinoma cell (HCC) [22], and prostate cancer cells, including PC-3 and LNCaP [23, 24]. Previous studies have shown that RSV-A2 can cause apoptosis, although their interactions were not fully understood.

The main objective of the present research was to investigate the impact of the oncolytic RSV-A2 on the proliferation of TC-1 cancer cell lines. We aimed to examine the relationship among RSV-A2-induced cytolysis, induction of apoptosis, ROS generation, autophagy, and cell cycle inhibition in vitro in the TC-1 cell line, which is used as a model for HPV-related cervical cancer cells. To evaluate the oncolytic dose of RSV-A2 in terms of cellular viability and metabolic activity, LDH and MTT tests were conducted using the ELISA technique. To evaluate apoptosis, ROS generation, autophagy, and cell cycle arrest, TC-1 tumor cell lines were infected with RSV-A2 and examined by flow cytometry, real-time PCR, and ELISA methods.

MTT and LDH are common approaches to assessing cell death. They were carried out to evaluate the oncolytic impact and titer of RSV-A2 in terms of metabolic rate and cell integrity. The MTT method was used to identify metabolic cells by determining the amount of formazan crystals formed through the reduction of tetrazolium salts by mitochondria. The LDH assay was employed to quantify intracellular lactate dehydrogenase leaking, a hallmark of cellular membrane breakdown and death of cells. Our LDH and MTT experiments indicated significant MOI-dependent toxicity of TC-1 cell lines following infection with RSV-A2. Consistently with the present findings, recent investigations have documented the oncolytic effects of RSV-A2 on cell proliferation in various human cancer cell lines, with no toxicity to normal cells. [20–24].

To investigate apoptosis as the oncolytic and antitumor mechanism of RSV-A2 on TC-1 cancer cells, flow cytometry was performed to detect annexin V/PI-stained apoptotic cells. Our findings revealed that the RSV-A2 oncolytic virus exerted its anticancer action via apoptosis. Annexin V/PI labeling verified the MOI-dependent engaging of apoptosis in TC-1 cell death induction compared to control cells. Recent experiments have investigated the mechanism by which RSV-A2 oncolytically destroys cancer cells. They have reported that RSV-A2 can inhibit the proliferation of certain types of tumor cells, such as skin [20], breast [21], and prostate [23, 24], cancer cells via triggering apoptosis.

Three types of cell death have been identified in multicellular organisms, namely apoptosis (Class I), autophagy (Class II), and necrosis (Class III), based on their morphologically distinctive features [30]. Apoptosis is an essential mechanism for eradicating infected or damaged cells to keep the integrity of tissues and cellular homeostasis. Two main apoptosis pathways (caspase-dependent) have been identified. They consist of mitochondrial (intrinsic) and death receptor (extrinsic) pathways [31]. Extrinsic apoptosis is activated by attaching death elements such as FAS-L (FAS ligand), TNF-α, and TRAIL (TNF-related apoptosis-inducing ligand) cytokine to FASR (FAS receptor), TNF-R1 (TNF receptor type1), TRAIL-R1, and TRAIL-R2 (TRAIL receptors types 1 and 2) receptors. These complexes of death receptors will stimulate caspases 8 and 10 [31]. The intrinsic pathway is triggered through the MOMP (mitochondrial outer membrane permeabilization) [32]. The MOMP induces cytochrome c leakage, which forms the apoptosome and activates caspase 9. Both routes eventually activate executioner caspases 3, 6, and 7, which cleave cytosolic and nuclear proteins [32]. Additionally, stimulation of caspase 8 by death receptor binding can activate the mitochondrial pathway through Bid. The induction of the mitochondrial pathway is thought to boost extrinsic apoptosis [31]. The Bcl2 family includes anti-apoptotic factors, such as Bcl2 and Bcl-XL (B-cell lymphoma-extra large), and proapoptotic elements, such as Bax and Bid (BH3 interacting-domain death agonist) [33]. It is believed that the proportions of proapoptotic and anti-apoptotic members define if cells survive or die because a rise in Bax to Bcl-2 results in the leakage of cytochrome C from mitochondria and induces the intrinsic pathway. [32, 34]. Autophagy is a cytosolic organelle degrading mechanism in lysosomes that has been correlated with homeostasis. Furthermore, autophagy has become an essential biological process in targeting human malignancies, such as cervical carcinoma. Historically, it has been related to non-apoptotic cellular death [35].

To study the underlying mechanism of apoptosis, gene expression of the Bcl-2 and Bax using real-time PCR assay, the protein level of TNF-α via ELISA method and activated caspase-3 value by flow cytometer were assessed in TC-1 cell lines after oncolytic RSV-A2 treatment. As depicted in Figs. 5 and 6, and 7, our results demonstrated that increasing infection doses could significantly decrease Bcl-2 expression, raise Bax mRNA level, and elevate TNF-α and cleaved caspase-3 protein levels versus control cells. We have demonstrated that RSV-A2 can induce apoptosis via caspase-dependent in TC-1 cells by activating both intrinsic and extrinsic pathways. In line with our findings, previous studies revealed that some oncolytic viruses can induce apoptosis via either intrinsic or extrinsic routes or both. [33, 34, 36].

Another indicator determined by flow cytometry was ROS generation following oncolytic RSV-A2 infection. Findings indicated that the RSV-A2 virus induces ROS formation in infected TC-1 cancer cells depending on the MOI in comparison with the control group. Increasing the Production of ROS can lead to cellular death, particularly in tumor cells that enhance the RSV-A2 oncolytic effectiveness. Prior research supported our findings that ROS generation might be implicated in apoptosis and autophagy in malignant cells [37–39]. There is evidence that producing reactive oxygen species causes death in cells via intrinsic apoptotic pathway by mitochondrial membrane breakdown, releasing Cytochrome-C into the cytoplasm and initiating apoptosis through ASK1/JNK (apoptosis -regulating kinase 1/ c-Jun N-terminal kinase) signaling [40]. ROS has been related to autophagic cellular death, a process in cells characterized by the development of autophagosomes and associated with Class II of programmed cell death. [37].

LC3B–II is a biomarker to measure cell autophagy rates. LC3B–II is present on the inside and outside of the autophagosome and is necessary for the growth and maturation of the autophagic membrane [41]. To examine autophagy as an additional oncolytic mechanism of the oncolytic RSV-A2 virus on TC-1 cancer cells, flow cytometry was applied to identify autophagosomes. Our findings demonstrated that RSV-A2 treatment of TC-1 cells could significantly increase LC3B- II level vs. the control group. Using flow cytometry to measure the value of LC3B-II, it was demonstrated that RSV-A2 induces autophagy. (Fig. 8). Along with our findings, several studies reported that autophagy had been linked to tumor inhibition [42]. In line with the current results, it has been shown that some oncolytic viruses and chemotherapy drugs can induce apoptosis and autophagy in cancer cells [14, 35, 43].

In order to further investigate the process causing RSV-A2’s inhibiting effect on TC-1 cells, we examined its impact on the progress of the cellular cycle. Thus, we followed the cell cycling phase distribution in RSV-A2 infected TC-1 cells, and subsequently, cell cycle development was analyzed by measuring the DNA content with flow cytometry. The results indicated that during 72 h of RSV-A2 infection, the percentage of cells in the proliferation G1 phase reduced significantly, while the number of cells in the sub-G1 (apoptotic), S, and G2M phases significantly increased. Findings demonstrate that RSV-A2 disrupts the progress of the cell cycle, leading to a cellular accumulation in the sub-G1, S, and G2 phases. Consistent with our research, recent studies have revealed that chemotherapeutic and oncolytic virus therapies in malignant cells can stop cells in various phases to inhibit tumor growth dose-dependently [32, 43–45].

## Conclusion

The results of this investigation indicate the cytotoxic effect of oncolytic RSV-A2 against the TC-1 tumor cell line of HPV-related cancer-producing human papillomavirus 16 (HPV-16) E6/E7 oncoproteins. It was shown that this virus caused anticancer activity through selective replication in cancerous cells, ROS generation, cell cycle arrest, stimulation of the intrinsic and extrinsic apoptotic pathways, and autophagy mechanisms. The findings offer new insight into the application of oncolytic RSV-A2 in in treating cervical cancer.

## Data Availability

The datasets used and analyzed during the current study are available from the corresponding author upon reasonable request.

## Abbreviations

OVs: Oncolytic viruses
ROS: Reactive oxygen species
HPV16: Human papillomavirus type 16
Bax: Bcl-2-associated X protein
Bcl2: B-cell leukemia/lymphoma 2 protein
Bcl-XL: B-cell lymphoma-extra large
BID: BH3 interacting-domain death agonist
IFN-1: Interferon type 1
T-VEC: Talimogene laherperepvec
HCC: Hepato cellular carcinoma
PC-3: Prostate cancer cell line 3
LNCaP: Lymph Node Carcinoma of the Prostate
E6/E7: Major transforming proteins of many types of papillomaviruses
FBS: Fetal bovine serum
HEPES: 4-(2-hydroxyethyl)-1-piperazineethanesulfonic acid
HEp-2: Human epithelial cell 2
Annexin V-FITC/PI: Annexin V-fluorescein isothiocyanate/propidium iodide
DCFH-DA: 2,7- dichlorofluorescein diacetate
qRT-PCR: Quantitative Real-Time PCR
BSA: Bovine serum albumin
T- Test: Student’s t-test
ANOVA: Analysis of variance
IC50: Inhibitory Concentration 50% growth
TRAIL: TNF-related apoptosis-inducing ligand
TNF-R1: TNF receptor type 1
TRAIL-R1 and R2: TRAIL receptors types1 and 2
FAS-L: FAS ligand
FAS-R: FAS receptor
MOMP: Mitochondrial outer membrane permeabilization
mRNA: messenger RNA
G1& G2 phases: Gap1 & 2 phases
S phase: Synthesis phase
RSV-A2: Respiratory Syncytial Virus A2 strain
DMEM: Dulbecco’s Modified Eagle Medium
CPE: Cytopathice effect
PFU: Plaque-forming units
BSA: Bovine serum albumin
LDH: Lactate dehydrogenase
ELISA: Enzyme-Linked Immunosorbent Assay
TNF-α: Tumor necrosis factor-α
cDNA: Complementary DNA
RT-qPCR: Reverse transcription-quantitative polymerase chain reaction
MOI: Multiplicity of infection
LC3B-II: Microtubule-associated Protein light chain 3B-II
pen/ strep: Penicillin/ streptomycin
HPI: hours post-infection
Ab: Antibody
PBS: Phosphate-buffered saline
RPM: Rotation per minute
ASK1/JNK: Apoptosis signal-regulating kinase 1/ c-Jun N-terminal kinase

## References

[1] WHO, Cervical cancer:, WHO. 2022. Available from: https://www.who.int/news-room/fact-sheets/detail/cervical-cancer.

[2] Sung H, Ferlay J, Siegel RL, Laversanne M, Soerjomataram I, Jemal A (2021). Global Cancer Statistics 2020: GLOBOCAN estimates of incidence and Mortality Worldwide for 36 cancers in 185 countries. Cancer J Clin.

[3] Lei J, Ploner A, Elfström KM, Wang J, Roth A, Fang F (2020). HPV Vaccination and the risk of Invasive Cervical Cancer. N Engl J Med.

[4] Stelzle D, Tanaka LF, Lee KK, Ibrahim Khalil A, Baussano I, Shah ASV (2021). Estimates of the global burden of cervical cancer associated with HIV. The Lancet Global Health.

[5] Keshavarz M, Nejad ASM, Esghaei M, Bokharaei-Salim F, Dianat-Moghadam H, Keyvani H (2020). Oncolytic Newcastle disease virus reduces growth of cervical cancer cell by inducing apoptosis. Saudi J Biol Sci.

[6] Han X, Wang S, Zhou W, Li Y, Lei WEN, Lv W (2015). Synergistic combination of histone deacetylase inhibitor suberoylanilide hydroxamic acid and oncolytic adenovirus ZD55-TRAIL as a therapy against cervical cancer. Mol Med Rep.

[7] X Wanga HM, Leea J, Xiea C (2022). Therapeutic implementation of oncolytic viruses for Cancer Immunotherapy: Review of Challenges and current clinical trials. J Biomedical Sci Res.

[8] Keshavarz M, Ebrahimzadeh MS, Miri SM, Dianat-Moghadam H, Ghorbanhosseini SS, Mohebbi SR et al. Oncolytic Newcastle disease virus delivered by mesenchymal stem cells-engineered system enhances the therapeutic effects altering tumor microenvironment. Virol J. 2020;17(1). 10.1186/s12985-020-01326-w PMC7201980 32370750

[9] Malhotra J, Kim ES (2022). Oncolytic viruses and Cancer Immunotherapy. Curr Oncol Rep.

[10] Lawler SE, Speranza M-C, Cho C-F, Chiocca EA (2017). Oncolytic viruses in Cancer Treatment. JAMA Oncol.

[11] Burton C, Bartee E (2019). Syncytia formation in Oncolytic Virotherapy. Mol Therapy - Oncolytics.

[12] Li X, Sun X, Wang B, Li Y, Tong J (2023). Oncolytic virus-based hepatocellular carcinoma treatment: current status, intravenous delivery strategies, and emerging combination therapeutic solutions. Asian J Pharm Sci.

[13] Dong H, Li M, Yang C, Wei W, He X, Cheng G et al. Combination therapy with oncolytic viruses and immune checkpoint inhibitors in head and neck squamous cell carcinomas: an approach of complementary advantages. Cancer Cell Int. 2023;23(1). 10.1186/s12935-022-02846-x PMC9814316 36604694

[14] Mozaffari Nejad AS, Fotouhi F, Mehrbod P, Keshavarz M, Alikhani MY, Ghaemi A (2020). Oncolytic effects of Hitchner B1 strain of newcastle disease virus against cervical cancer cell proliferation is mediated by the increased expression of cytochrome C, autophagy and apoptotic pathways. Microb Pathog.

[15] Chang J (2011). Current progress on development of respiratory syncytial virus vaccine. BMB Rep.

[16] Mejias A, Rodríguez-Fernández R, Oliva S, Peeples ME, Ramilo O (2020). The journey to a respiratory syncytial virus vaccine. Ann Allergy Asthma Immunol.

[17] Drysdale SB, Barr RS, Rollier CS, Green CA, Pollard AJ, Sande CJ. Priorities for developing respiratory syncytial virus vaccines in different target populations. Volume 12. Science Translational Medicine |REVIEW; 2020. 10.1126/scitranslmed.aax2466 PMC7613568 32188721

[18] Tregoning JS, Schwarze (2010). Respiratory viral infections in infants: causes, clinical symptoms, Virology, and Immunology. Clin Microbiol Rev.

[19] Domachowske JB, Anderson EJ, Goldstein M (2021). The future of respiratory Syncytial Virus Disease Prevention and Treatment. Infect Dis Therapy.

[20] Salimi V, Yaraki MT, Mahmoodi M, Shahabi S, Gharagozlou MJ, Shokri F (2013). The Oncolytic Effect of Respiratory Syncytial Virus (RSV) in human skin Cancer cell line, A431. Iran Red Crescent Med J.

[21] al. Ze. Breast Cancer Therapy Using an Engineered Respiratory Syncytial Virus. United States Patent. 2013.

[22] Choi SH, Park BK, Lee K-W, Chang J, Lee Y, Kwon H-J (2015). Effect of respiratory syncytial virus on the growth of hepatocellular carcinoma cell-lines. BMB Rep.

[23] Echchgadda I, Kota S, DeLa Cruz I, Sabbah A, Chang T, Harnack R (2009). Anticancer oncolytic activity of respiratory syncytial virus. Cancer Gene Ther.

[24] Echchgadda I, Chang T-H, Sabbah A, Bakri I, Ikeno Y, Hubbard GB (2011). Oncolytic targeting of androgen-sensitive prostate tumor by the respiratory syncytial virus (RSV): consequences of deficient interferondependent antiviral defense. BMC Cancer.

[25] Kagabu M, Yoshino N, Saito T, Miura Y, Takeshita R, Murakami K (2020). The efficacy of a third-generation oncolytic herpes simplex viral therapy for an HPV-related uterine cervical cancer model. Int J Clin Oncol.

[26] Leddon JL, Chen C-Y, Currier MA, Wang P-Y, Jung FA, Denton NL (2014). Oncolytic HSV virotherapy in murine sarcomas differentially triggers an antitumor T-cell response in the absence of virus permissivity. Mol Therapy - Oncolytics.

[27] Wang Y, Li Y, Dong S et al. Jing Jin1, Dong5 Y, Liu3 F,. Stability and anti-tumor effect of oncolytic herpes simplex virus type 2. Oncotarget,. 2018;9(37):24672-83. 10.18632/oncotarget.25122 PMC5973869 29872496

[28] Hassan SAH, Allawe AB, Al-Shammari AM (2020). In Vitro oncolytic activity of non-virulent Newcastle Disease Virus LaSota strain against mouse mammary adenocarcinoma. Iraqi J Sci.

[29] Russell L, Peng K-W (2018). The emerging role of oncolytic virus therapy against cancer. Chin Clin Oncol.

[30] Jung S, Jeong H, Yu S-W (2020). Autophagy as a decisive process for cell death. Exp Mol Med.

[31] Sun S-Y, Yue P, Zhou J-Y, Wang Y, Choi Kim H-R, Lotan R (2001). Overexpression of Bcl2 blocks TNF-Related apoptosis-inducing ligand (TRAIL)-Induced apoptosis in Human Lung Cancer cells. Biochem Biophys Res Commun.

[32] Kalyanasundram J, Hamid A, Yusoff K, Chia SL (2018). Newcastle disease virus strain AF2240 as an oncolytic virus: a review. Acta Trop.

[33] Mohammadian J, Shanehbandi, Samadi N, Sabzichi M, Molavi O (2016). Dariush. Combined treatment with Stattic and Docetaxel alters the Bax/ Bcl-2 gene expression ratio in human prostate Cancer cells. Asian Pac J Cancer Prev.

[34] Pereira Soares NdC, Teodoro AJ, Oliveira FL, Takiya CM, Junior AP, Nasciutti LE (2014). Lycopene induce apoptosis in human prostate cells and alters the expression of bax and Bcl-2 genes. LWT - Food Science and Technology.

[35] Li S, Li Z, Chen S, Zhu Y, Li Y, Yin X (2022). Apoptotic and autophagic cell death induced in cervical cancer cells by a dual specific oncolytic adenovirus. Anticancer Drugs.

[36] Doan P, Musa A, Candeias NR, Emmert-Streib F, Yli-Harja O, Kandhavelu M. Alkylaminophenol induces G1/S phase cell cycle arrest in Glioblastoma cells through p53 and cyclin-dependent kinase signaling pathway. Front Pharmacol. 2019;10. 10.3389/fphar.2019.00330 PMC6454069 31001122

[37] Beth Levine1 aDJK (2004). Development by Self-Digestion: Molecular Mechanisms and Biological Functions of Autophagy. Dev Cell.

[38] Hui G, Yu LL, Quan ZMaZ (2016). Inactivated Sendai Virus induces apoptosis mediated by reactive oxygen species in murine melanoma cells. Bio med Environ Sci.

[39] Han Z, Li Q, Sun S, Zhao W, Shi L (2018). Inactivated Sendai virus strain Tianjin induces apoptosis and autophagy through reactive oxygen species production in osteosarcoma MG-63 cells. J Cell Physiol.

[40] Salehi F, Behboudi H, Kavoosi G, Ardestani SK (2017). Chitosan promotes ROS-mediated apoptosis and S phase cell cycle arrest in triple-negative breast cancer cells: evidence for intercalative interaction with genomic DNA. RSC Adv.

[41] Glick D, Barth S, Macleod KF (2010). Autophagy: cellular and molecular mechanisms. J Pathol.

[42] Jin S (2014). p53, autophagy and tumor suppression. Autophagy.

[43] Yao G, Chen H, Chen L, Ge M, Yang J, Liu W (2017). Autophagy promotes apoptosis induction through repressed nitric oxide generation in the treatment of human breast cancer MCF-7 cells with L-A03, a dihydroartemisinin derivative. Med Chem Res.

[44] babaei A, Soleimanjahi H, Arefian E. Oncolytic reovirus shows potentially anticancer effect against CT26 cell lines 2019.

[45] Igase M, Shibutani S, Kurogouchi Y, Fujiki N, Hwang CC, Coffey M (2019). Combination therapy with Reovirus and ATM inhibitor enhances cell death and virus replication in Canine Melanoma. Mol Therapy - Oncolytics.

